# Impact of Bone Morphogenetic Protein 7 and Prostaglandin receptors on osteoblast healing and organization of collagen

**DOI:** 10.1371/journal.pone.0303202

**Published:** 2024-05-16

**Authors:** Mohammad Ali Salama, Asmaa Anwar Ismail, Md Sofiqul Islam, Aghila Rani K. G., Sausan Al Kawas, A. R. Samsudin, Smriti Aryal A. C.

**Affiliations:** 1 Department of Oral and Craniofacial Health Sciences, College of Dental Medicine, University of Sharjah, Sharjah, United Arab Emirates; 2 Research Institute for Medical and Health Sciences, University of Sharjah, Sharjah, United Arab Emirates; 3 Department of Operative Dentistry, RAK College of Dental Sciences, RAK Medical and Health Sciences University, Ras Al Khaimah, United Arab Emirates; King Faisal University, SAUDI ARABIA

## Abstract

**Purpose:**

This study seeks to investigate the impact of co-administering either a Prostaglandin EP2 receptor agonist or an EP1 receptor antagonist alone with a low dose BMP7 on in vitro healing process, collagen content and maturation of human osteoblasts.

**Methodology:**

Human osteoblast cells were used in this study. These cells were cultured and subjected to different concentrations of Prostaglandin EP2 receptor agonist, EP1 receptor antagonist, BMP7, Control (Ct) (Vehicle alone), and various combinations treatments. Cell viability at 24, 48 and 72 hours (h) was evaluated using the XTT assay. A wound healing assay was conducted to observe the migration ability of human osteoblast cells. Additionally, Sirius red staining and Fourier-Transform Infrared Spectroscopy Imaging (FT-IR) was employed to analyze various parameters, including total protein concentration, collagen production, mature collagen concentration, and mineral content.

**Results:**

The combination of low dose BMP7 and Prostaglandin EP2 receptor agonist resulted to the lowest cell viability when compared to both the Ct and individual treatments. In contrast, the Prostaglandin EP1 receptor antagonist alone showed the highest cellular viability at 72 h. In the wound healing assay, the combined treatment of low dose BMP7 with the Prostaglandin EP2 receptor agonist and EP1 receptor antagonist showed a decrease in human osteoblast healing after 24 h. Analysis of FT-IR data indicated a reduction in total protein content, collagen maturity, collagen concentration and mineral content in combination treatment compared to the single or Ct treatments.

**Conclusion:**

The combination of a Prostaglandin EP2 receptor agonist or an EP1 receptor antagonist when combined with low dose BMP7 significantly hinders both human osteoblast healing and collagen maturity/concentration in comparison to low dose BMP7 treatment alone.

## Introduction

Bone tissue is a dynamic tissue, continually undergoing remodeling characterized by cycles of destruction and reconstruction [1, 2]. The term used for this process is called remodeling, a process primarily regulated by osteoclasts, which resorbs the bone and osteoblasts, involved in new bone formation [3]. Bone tissues are among the few tissues that are capable of regeneration, in which the healed bone is structurally identical to the adjacent bone. Despite its regenerative capacity, impediments to the healing process can arise due to factors such as aging, metabolic diseases, and smoking. These elements can result in delayed healing or non-union in 10–20% of fractures, resulting in considerable disability [4]. One of the prominent challenges facing contemporary society is the need to address the increasing demand for revision surgeries for fractured bones experiencing non-union [5]. Additionally, there is a notable difficulty in facilitating bone regeneration among elderly individuals with dental implants, leading to heightened morbidity and health-related risks [6]. PGE2 (prostaglandin E2), an anabolic non-peptide agent, has been implicated to stimulate bone formation in both systemic administrations to animals and human beings, as well as local application for improving bone formation and healing in animals [7–9] serving as a crucial inflammatory mediators, that are required in facilitating healing process and contributing a significant role in bone biology. Nevertheless, PGE2, on the other hand presents notable systemic side effects, requiring multiple injections over several weeks to induce bone growth [8, 9].

The anabolic effect of PGE2 on target cells is facilitated through G protein–coupled receptor subtypes, namely EP1, EP2, EP3, and EP4. PGE2 exerts its influence by binding to four receptors: EP1- 4 [10–12]. While both EP2 and EP4 receptors exhibit both bone anabolic and catabolic effects [13–17], the EP4 receptor is predominantly expressed in osteoblasts [10, 11]. Limited information is available regarding the role of the EP1 receptor in bone biology; however, research suggests that EP1 enhances the proliferation of osteoblastic cell-line [18].

Bone morphogenetic proteins (BMPs) have demonstrated osteogenic potential by promoting bone formation via two distinct ways. They attract and mobilize mesenchymal stem cells from nearby surrounding muscle, bone marrow, and blood vessels. Subsequently, these recruited cells undergo differentiation, developing into osteoblasts to directly contribute to bone formation or transforming into cartilage cells which then mature into bone cells. Additionally, BMPs play a vital role in supporting the formation of bone matrix and stimulating vascularization. In vivo during bone repair, multiple BMPs are naturally expressed. For non-union fractures, BMPs are applied to stimulate healing in cases where previous attempts have failed whereas in acute fractures, BMPs are utilized to expedite the healing process and reduce the necessity for subsequent therapeutic interventions [19–22].

In the routine clinical setting, difficulties in managing disrupted or delayed fracture healing persist among the healthcare practitioners, additionally critical bone defects arising from trauma, infection, pseudarthrosis or tumor removal may necessitate the replacement of bone tissue. Today’s gold standard for treating significant bone abnormalities is autologous bone transplantation. Due to limitations in autologous bone transplantation and its associated drawbacks, new options to replace bone and osteo-promotive or osteo-inductive materials are being investigated in a variety of ways. One of the factors used to improve bone regeneration clinically are growth factors. Among the growth factors, Bone morphogenetic proteins (BMPs) stands out as an important one. These proteins have demonstrated osteogenic activity in animal study and have been used to induce bone formation in human [19].

Nevertheless, BMP’s morphogenic activity has been linked to substantial ectopic bone growth, leading to potential nerve injury in the spinal column including issues with bladder malfunction have been reported [23, 24]. Additionally, it is widely recognized that the process of bone formation demands a comparatively substantial amount of Bone Morphogenetic Proteins (BMPs), particularly in primates as opposed to rodents. However, one of the major challenges in human bone regeneration lies in the cost or expenses associated in using recombinant proteins when they are administered in large quantities along with its negative effects when used in high doses [25, 26]. Due to the significant shortcomings and high failure rates with existing techniques, there is an urgent demand or need for innovative approaches to enhance or stimulate bone formation, accelerate fracture healing, and treat non-unions that is why combining low-dose BMPs with other anabolic agents emerges as a cost-effective and advantageous solution beneficial to society.

This study aims to examine the effects of combining an EP2 receptor agonist or an EP1 receptor antagonist with BMP7 on human osteoblast healing and collagen maturation in vitro. Our hypothesis suggests that the treatment involving Prostaglandin EP2 receptor agonist or EP1 receptor antagonist coupled with BMP7 significantly influences the organization of the osteoblast collagen matrix and the healing of human osteoblasts, compared to treatment with BMP7, EP2 receptor agonist or EP1 receptor antagonist alone.

## Materials and methods

### Cell culture

Fetal Human osteoblasts (HOB) from Addex-bio, USA were cultured and consistently sustained in Dulbecco’s Modified Eagle’s medium DMEM / F12 Ham (Sigma, USA). The DMEM/ F12 media was supplemented with 10% Fetal Bovine Serum (FBS) (Sigma, USA) and 1% penicillin/streptomycin (Sigma, USA). The cell culture was conducted in a humidified chamber at 37°C with 5% CO_2_.

### Cell viability assay

HOB was cultured in T-75 cm^2^ flasks, washed twice with 1x PBS, detached using 1X trypsin EDTA (Sigma, USA), and centrifuged at 1200 RPM, 5 minutes at room temperature. Following centrifugation, the supernatant was discarded, the pellet was suspended in 1 mL complete DMEM/ F12 media. Then a 10 μL of the HOB cell suspension was mixed with 10 μL of trypan blue then resuspended thoroughly for quantification of HOB cell number. HOB was then plated in triplicates at a seeding density of 1x10^4^ cells per well in 96-well cell culture plates in a final volume of 200 μL of complete DMEM/ F12 media. After 24 h, HOB were treated with Prostaglandin EP1 receptor antagonist alone (100 ng/mL) (HY-108563/CS-0029175, Med Chem Express, USA), EP2 receptor agonist alone (100 ng/mL) (HY-14839/CS-5572, Med Chem Express, USA), Dimethyl sulfoxide (DMSO) vehicle as Ct, human Bone morphogenic protein 7 alone (BMP7) (50 ng/mL)(cyt-333-b, PROSPEC Bio, USA), combination of EP1 receptor antagonist (100 ng/ml) with BMP7 (50 ng/ml) or combination of EP2 receptor agonist (100 ng/ml) with BMP7 (50 ng/ml) for 24, 48, and 72 h respectively. At the end of each timepoint, images of the treated cells at each concentration were captured using the Olympus cell Sens imaging software (Olympus Life Science, Tokyo, Japan). After each timepoint, the treatment was discarded, and the cells were incubated for 4 h at dark with 100 μL/ well of the XTT reagent (Cell Proliferation Kit II, Roche, UK) to determine the percent cell viability post treatment. After the incubation period, the optical density reflecting the number of viable cells capable of cleaving the tetrazolium salts was determined using the Bio-Tek 800 TS microplate reader (Bio Tek, USA) at 450 nm.

### Wound healing assay

The wound healing assay utilized the wound healing assay kit (Abcam, UK) in accordance with manufacturer’s guidelines. In summary, the inserts were positioned in the plates to create a uniform wound field in a specific direction, ensuring secure contact with the plate well bottom. A 500 μL HOB cell suspension (7.5x10^4^/ well) was gently added through the insert’s open end at the top, with an additional 250 μL on either side for optimal cell dispersion. The plates were then incubated in a cell culture incubator until a monolayer formed. Following incubation, the inserts were carefully removed, and the culture wells were washed with 1X PBS to eliminate dead cells and debris. HOB cells were treated accordingly as mentioned in Table 1. in complete DMEM /F12 media supplemented with 2.5% FBS. Subsequently, HOB cells were allowed to migrate for 24 h. Wound closure was observed using a light microscope, and images were captured both time zero and 24 h using Olympus cell Sens imaging software (Olympus Life Science, Tokyo, Japan). The percentage closure or migration rate of HOB cells into the wound field was then analyzed using Image J Software (National Institutes of Health, USA).

**Table 1:** List of names of the groups and drugs dose used.

#	Name of groups with treatment and dose used
**1**	Control (Ct) group—DMSO vehicle treatment only
**2**	Human Bone Morphogenetic Protein 7 (BMP7) treatment alone (50 ng/ml)
**3**	Prostaglandin EP1 receptor antagonist treatment alone (100 ng/ml)
**4**	Combination dose EP1 receptor antagonist (100 ng/ml) with BMP7 treatment (50 ng/ml)
**5**	Prostaglandin EP2 receptor agonist treatment alone (100 ng/ml)
**6**	Combination dose EP2 receptor agonist (100 ng/ml) with BMP7 treatment (50 ng/ml)

This Table provides a concise description of the name of the experimental groups and type of treatments and the doses used in each group.

### Collagen organization/maturation by picrosirius red (PSR) staining

HOB was seeded at the density of 7 × 10^4^ cells/well in coverslips in 6 well plates. The medium was replaced with DMEM/ F12 media containing 50 μg/mL ascorbic acid after 24 h of plating and then treated with different treatment groups mentioned in Table 1. The cells were incubated for a period of 21 days, with a medium change occurring every 2–3 days. Prior to staining, HOB cell culture was washed twice with 1x PBS and fixed with 4% PFA for 30 minutes. Direct red stain (Sigma, USA) was prepared at a concentration of 1 mg /mL dissolved in picric acid. HOB cells after fixation with PFA and washed twice with 1X PBS then incubated for 30 minutes at room temperature with 1 mL/ well of the prepared direct red stain. Collagen fibers stained in red were captured using the Olympus cell Sens imaging software (Olympus Life Science, Tokyo, Japan). After imaging, cells were washed with 1M acetic acid to remove the unbound stain and eluted in 1 mL of de-staining solution composed of 0.2 M NaOH/methanol 1:1 and the absorbance was measured at 570 nm on a microplate reader.

### Osteogenic media preparation

To induce the osteogenic differentiation of HOB, incomplete DMEM/F12 media was supplemented with 10 nM dexamethasone (Sigma, USA), 10 mM β-glycerophosphate (Sigma, USA), and 50 μg/mL ascorbic acid (Sigma, USA) [27]. After adding these components, the media was filtered using a sterile 0.2 μm syringe filter (Thermo-Fisher Scientific, USA) to eliminate any microbial contamination. 10% FBS and 1% penicillin/streptomycin were then added to the media.

### Alizarin red staining and calcium release assay

Alizarin Red (Sigma-Aldrich, USA) stain was prepared by weighing 680 mg of Alizarin Red S powder and dissolving it in 50 mL of distilled water. The pH was then adjusted to 4.1 and the solution was filtered using 150 mm Whatman filter papers. HOB was seeded at 5x10^4^ seeding density in 12-well culture plates and incubated for 24 hours. After a monolayer formed, cells were treated with the various treatments mentioned in Table 1. which were prepared in osteogenic induction media. Media and treatment were replaced every 3–4 days until 21 days of culture. After 21 days, the cells were washed twice with DPBS and then fixed using 4% PFA at room temperature for 30 minutes. Cells were then washed twice with DPBS and 1 mL of the alizarin red stain was added to the cells [28]. Cells were then incubated 30 minutes on a rocking platform to allow the stain to detect the presence of calcium release. After the incubation, the cells were washed twice with distilled water and the stained nodules were captured using the Olympus cell Sens imaging software (Olympus Life Science, Tokyo, Japan). For quantification, 800 μL of 10% acetic acid was added to the stained cells and the plate was incubated for 30 minutes at room temperature with shaking [29]. The cells were then collected using a cell scraper, transferred into 1.5 mL microcentrifuge tubes, and vortexed vigorously. The cell suspensions were then kept in a heat block at 85°C for 10 minutes to allow the cells to release their stain. The suspensions were then incubated on ice for 5 minutes and centrifuged at 12,000 RPM for 15 minutes. 500 μL of the cell suspensions were transferred into new tubes and 200 μL of NH_4_OH were added to each tube to neutralize the acid. 150 μL of the samples were then transferred into a 96-well plate to determine the optical density at 405 nm using the Bio-Tek 800 TS microplate reader.

### Analysis of mineral and protein by Fourier-Transform Infrared Spectroscopy (FT-IR) imaging

HOB cells were seeded onto coverslips in tissue culture treated dishes at a density of 7 × 10^4^ in osteogenic medium, as described above. About 48 h later, osteogenic media was replaced with media containing either no treatment or treatments with Prostaglandin EP2 receptor agonist alone, EP1 receptor antagonist alone, BMP7 alone, DMSO vehicle alone (Ct) and combination of Prostaglandin EP2 receptor agonist with BMP7 or Prostaglandin EP1 receptor antagonist with BMP7. Subsequently, the cultures were returned to the incubator, with media and treatments refreshed every 3–4 days until reaching a 21-day culture period. On day 21, media was discarded, and the cultures were rinsed twice with 1x PBS. FT-IR data were then collected in transmittance within the mid-IR spectral region, utilizing FT-IR- 6300 spectrometer (Jasco, Japan) with a spatial resolution of 50 μm. The images derived from the infrared spectra were subjected to analysis using dedicated spectra analysis software. Evaluation of total protein, mineral contents and the collagen were assessed based on the integrated areas [30].

### Statistical analysis

The entire dataset was collected, organized, and underwent comprehensive statistical analysis by SPSS software (version 24, Chicago, Illinois). This involved initial assessment for normality using the Shapiro-Wilk test, guiding the choice between parametric and non-parametric tests, with a preference for parametric tests when normality was observed. Parametric testing involved the application of One-way ANOVA with subsequent post hoc Tukey analysis for multiple comparisons. Significance was established at P <0.05, with a higher threshold set at P <0.01. Additionally, non-parametric testing involved the use of Kruskal-Wallis One-way ANOVA. Despite using different approaches, both non-parametric and parametric tests yielded similar results. Graphs illustrating the data were generated using GraphPad Prism software (CA, USA).

## Results

### Cell viability

HOB cells were divided into 6 experimental groups as mentioned below in Table 1.

XTT cell viability assay was done to check the HOB cells viability under different treatment conditions after 24, 48 and 72 h. All the groups demonstrated a significant increase in HOB cell viability across different timepoints (24, 48 and 72h) (p<0.05). When the HOB cellular viability was compared, it was observed that the viability of the HOB treated with Ct was higher than that of all the other groups. However, this difference was only statistically significant when compared to the group treated with the combination of BMP7+EP2 receptor agonist (p<0.01). The group with the lowest HOB cell viability, in comparison to all other groups, was the one treated with the combination of BMP7+ EP2 receptor agonist, and this difference observed was significant when contrasted with the other treatment groups (p<0.01).

At the 24 h timepoint, the Ct group exhibited the highest HOB cell viability, and the observed distinction achieved statistical significance in comparison to all other groups (p<0.01).The treatment group with the lowest HOB cell viability among the various treatments groups was the one receiving the combination of BMP7+EP2 receptor agonist; Nevertheless, no substantial difference was noted between this particular group and the other treatment groups (p>0.05).

At the 48 h timepoint, a statistically significant increase in cellular viability was noted in all groups (p<0.05). While the group treated solely with the EP2 receptor agonist exhibited the highest HOB cellular viability compared to other groups however, the difference was not significant (p>0.05). Conversely, the group with the combination of BMP7+ EP2 receptor agonist exhibited the lowest HOB cellular viability, and the observed disparity exhibited significance when compared to all other treatment groups (p<0.05).

**Fig 1:**
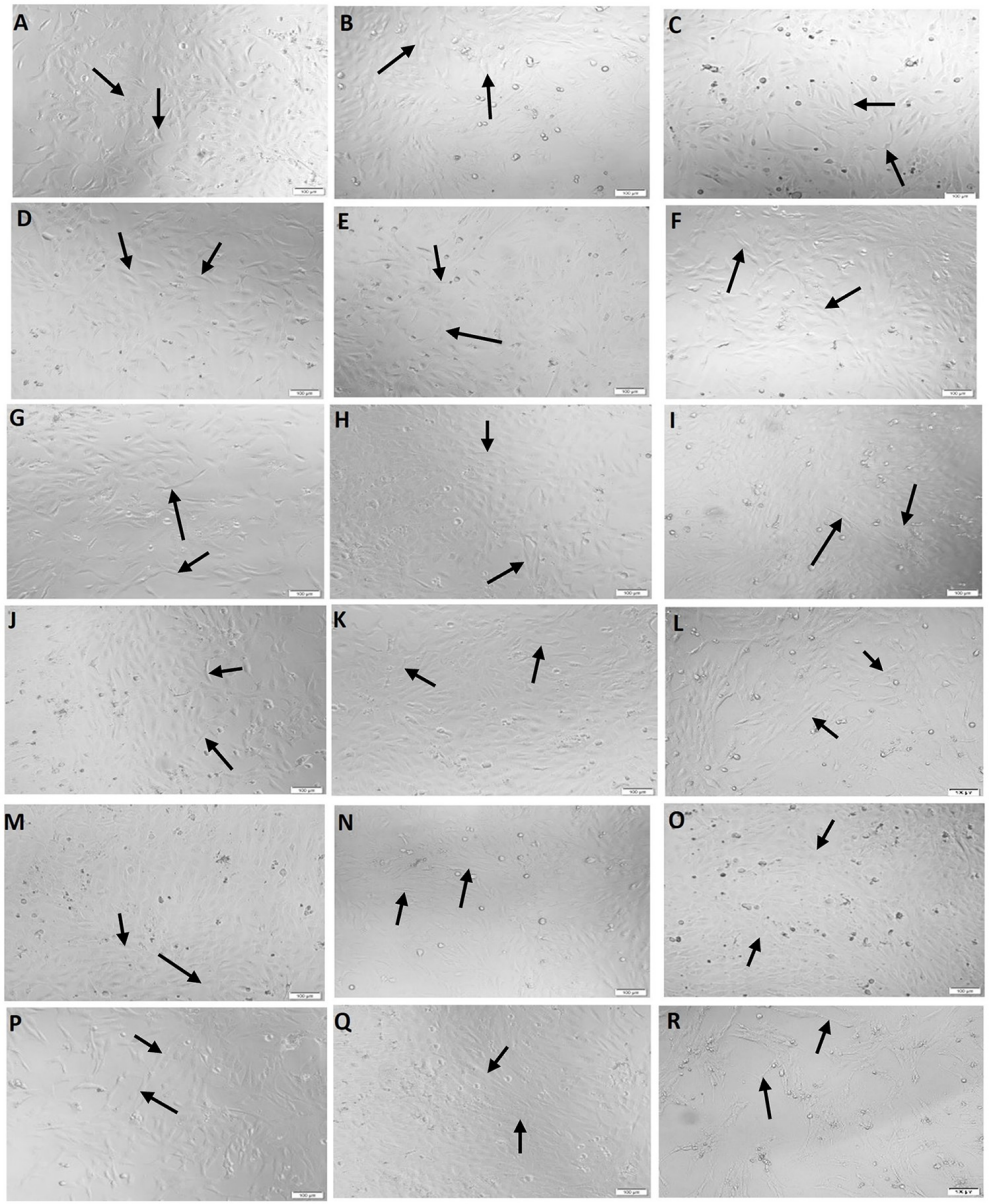
Representative light microscopy images showing HOB cells morphology at 24, 48, 72 h respectively of treatment with **(A-C)** Ct (DMSO vehicle treatment); **(D-F)** BMP7 alone (50 ng/ml); **(G-I)** EP1 alone (100 ng/ml); **(J-L)** BMP7 (50 ng/ml) with EP1 (100 ng/ml); **(M-O)** EP2 alone (100 ng/ml); **(P-R)** BMP7 (50 ng/ml) with EP2 (100 ng/ml). The black arrows indicate the viable cells. Scale bar 100 μm.

**Fig 2:**
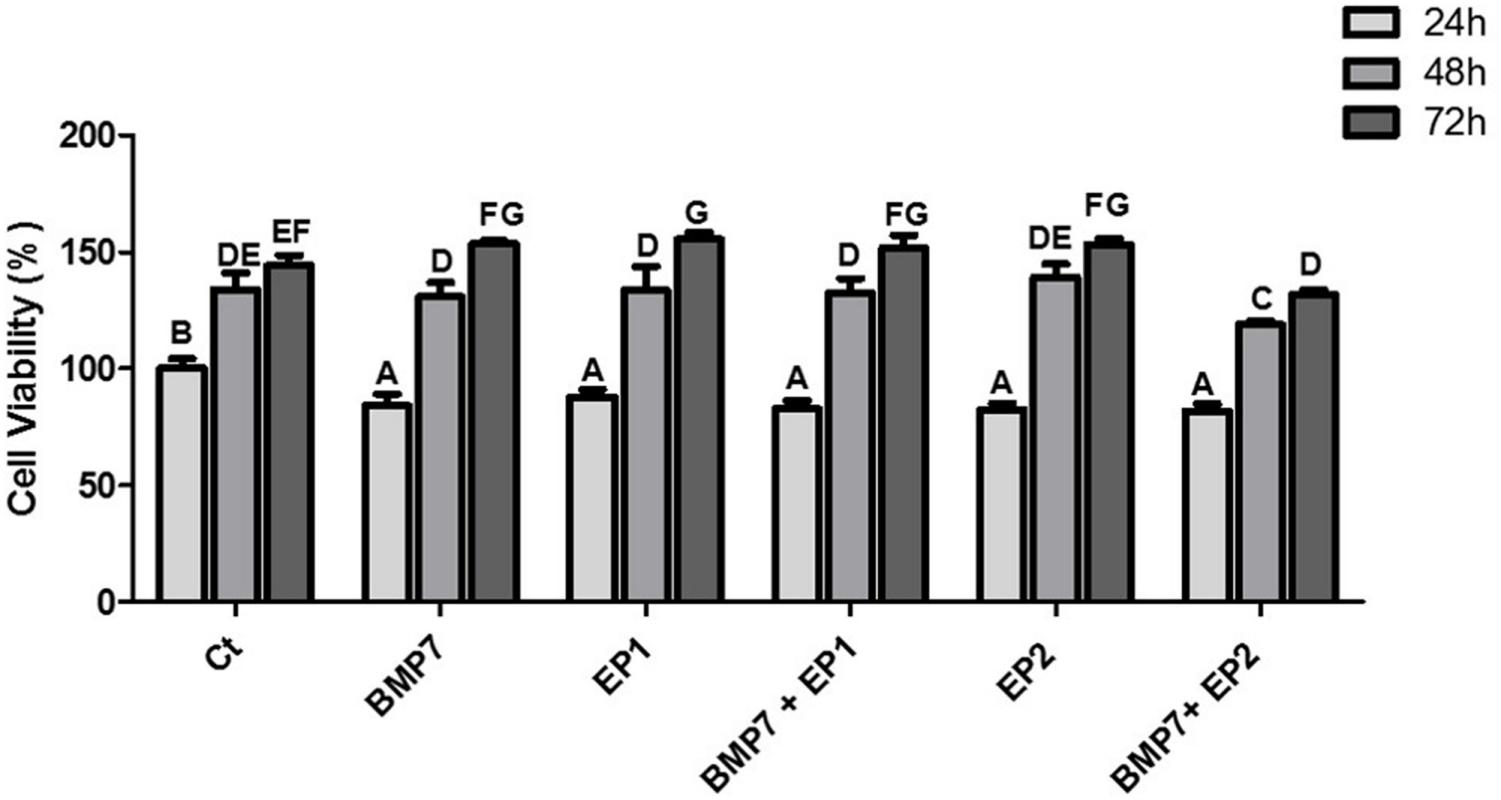
The graph represents the viability of HOB cells in response to treatment with Ct (DMSO vehicle treatment); BMP 7 alone (50 ng/ml); EP1alone (100 ng/ml); BMP7 (50 ng/ml) with EP1 (100 ng/ml); EP2 alone (100 ng/ml); BMP7 (50 ng/ml) with EP2 (100 ng/ml) at three time points: 24, 48 and 72 h respectively. The findings are reported as mean ± standard deviation, and statistical analysis was performed using ANOVA. Groups sharing identical alphabets above each bar indicate no significant statistical differences.

At the 72 h timepoint, there was a statistically notable rise in HOB cell viability in all groups when compared to the corresponding treatment groups at 48 h time point. The treatment group exhibiting the highest HOB cellular viability was the one exclusively treated with the EP1 receptor antagonist; however, the difference reached statistical significance only when compared to the Ct group and the treatment group receiving the combination of BMP7+EP2 receptor agonist (p<0.05). However, the group with the lowest HOB cell viability was the one treated with the combination of BMP7+EP2 receptor agonist, although this difference did not show statistical significance (p>0.05). All light microscopy pictures and cell viability assay graphs are shown in Figs 1 & 2 respectively.

### Wound healing

**Fig 3:**
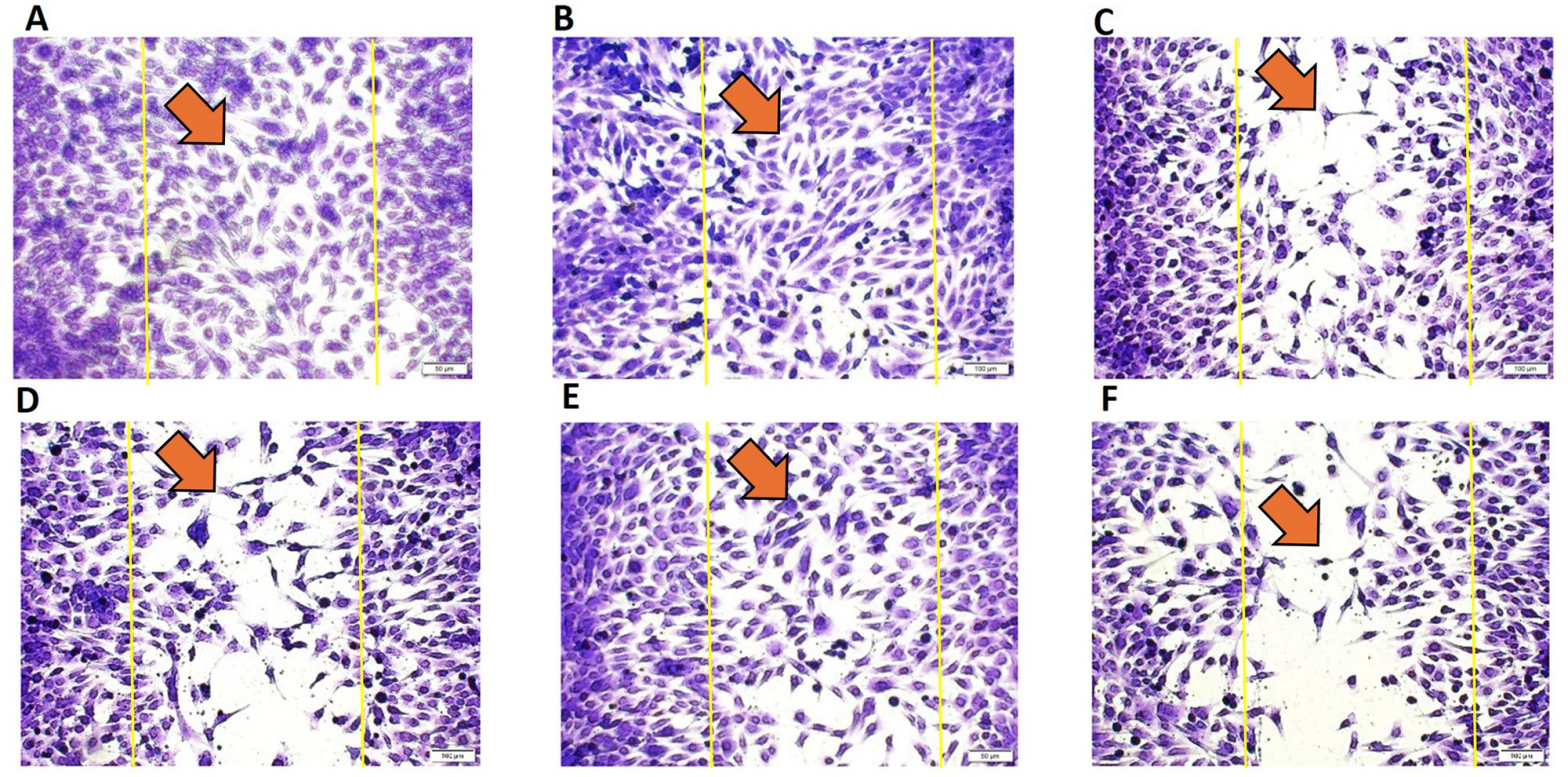
Light microscopy images illustrating the quantitative gap percentage following the induction of scratch or wounds in human osteoblast cells monolayer. The wound area is depicted by two yellow vertical lines, with an orange arrow pointing to the migrating cells. The cells were treated with **(A)** Ct (DMSO vehicle treatment only), **(B)** BMP7 (Bone morphogenic protein 7), **(C)** EP1 (EP1 receptor antagonist group), **(D)** BMP7+EP1 (the combination treatment of BMP7 with EP1 receptor antagonist), **(E)** EP2 (EP2 receptor agonist group), **(F)** BMP7+EP2 (the combination treatment of BMP7 with EP2 receptor agonist) after a duration of 24 h. Scale bar 100 μm.

**Fig 4:**
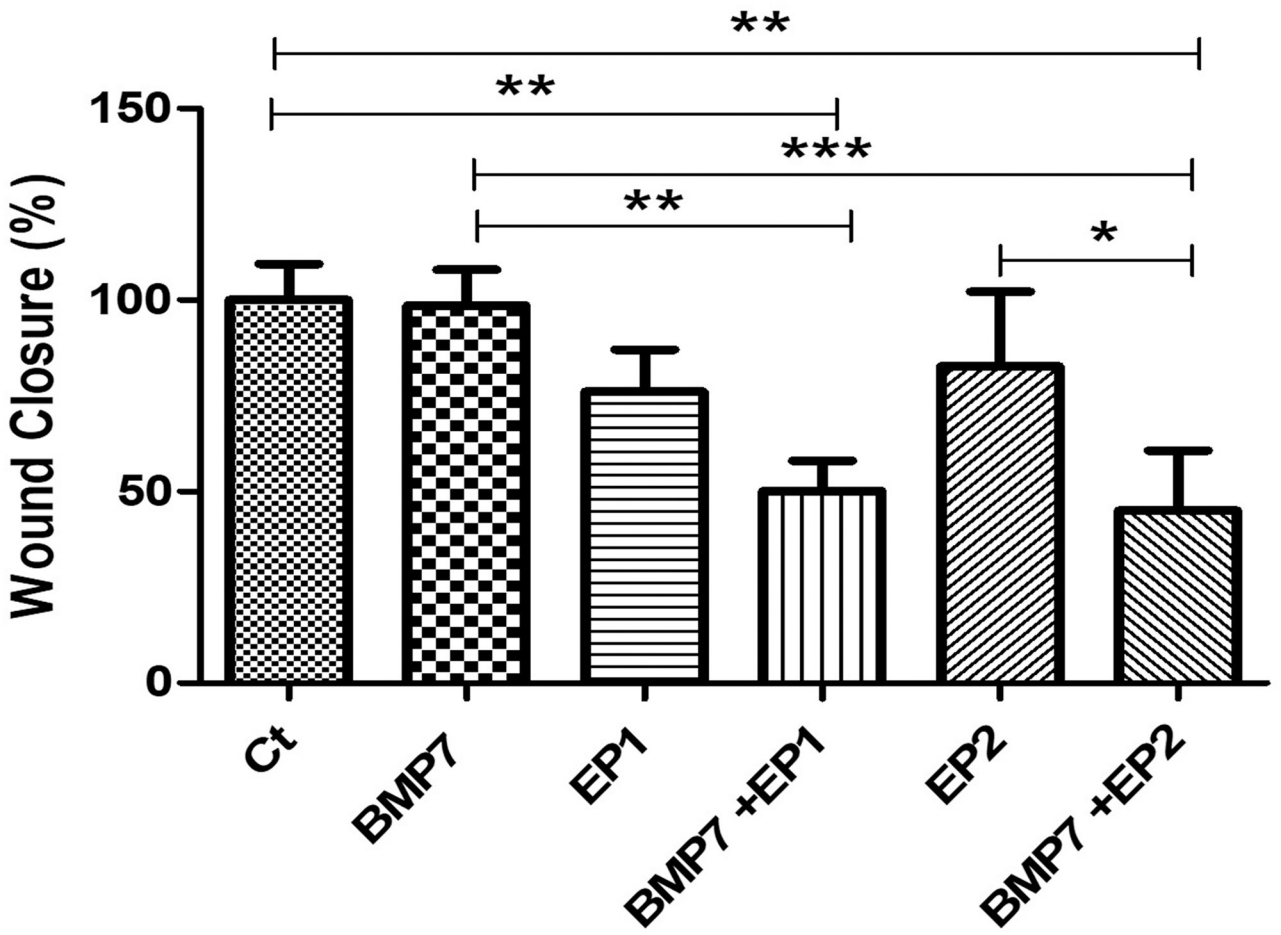
Graph showing the wound closure % rate or wound closure of human osteoblast cells under treatment with Ct (DMSO vehicle treatment only), BMP7 (Bone morphogenic protein 7), EP1 (EP1 receptor antagonist group), EP2 (EP2 receptor agonist group), BMP7+EP1 (the combination treatment of BMP7 with EP1 receptor antagonist), BMP7+EP2 (the combination treatment of BMP7 with EP2 receptor agonist) after a duration of 24 h. The results are expressed as mean ± standard deviation, and statistical analysis was carried out using ANOVA. Groups sharing identical alphabets above each bar indicate no significant statistical differences.

A scratch wound healing model [31] was established to access HOB cell migration ability in response to various treatments. The cellular migration was measured after creating a scratch in the HOB cell monolayer across different treatment groups, including Ct (DMSO vehicle treatment); BMP7 alone (50 ng/ml); EP1 alone (100 ng/ml); BMP7 (50 ng/ml) with EP1 (100 ng/ml); EP2 alone (100 ng/ml); BMP7 (50 ng/ml) with EP2 (100 ng/ml). The percentage of wound closure normalized to the Ct group was determined 24 h post-treatment. The Ct group exhibited the highest migration (wound closure rate) but this difference was not statistically significant when compared to BMP7, EP1 receptor antagonist alone, and EP2 receptor agonist alone (p>0.05). Significance was observed only when comparing the Ct group to the combination treatments of BMP7 with EP1 receptor antagonist and BMP7 with EP2 receptor agonist (P<0.01). The combination treatment groups showed the least amount of migration, when comparing the groups treated with EP1 receptor antagonist alone and the combination of BMP7 with EP1 receptor antagonist alone, However, no statistically significant difference was observed (p>0.05). Nevertheless, the combination treatment of BMP7 with EP2 receptor agonist showed a significantly lower percentage of wound closure compared to EP2 receptor agonist alone (P<0.05). All the light microscopy pictures and graphs are shown in Figs 3 & 4 respectively.

### Sirius red staining

**Fig 5:**
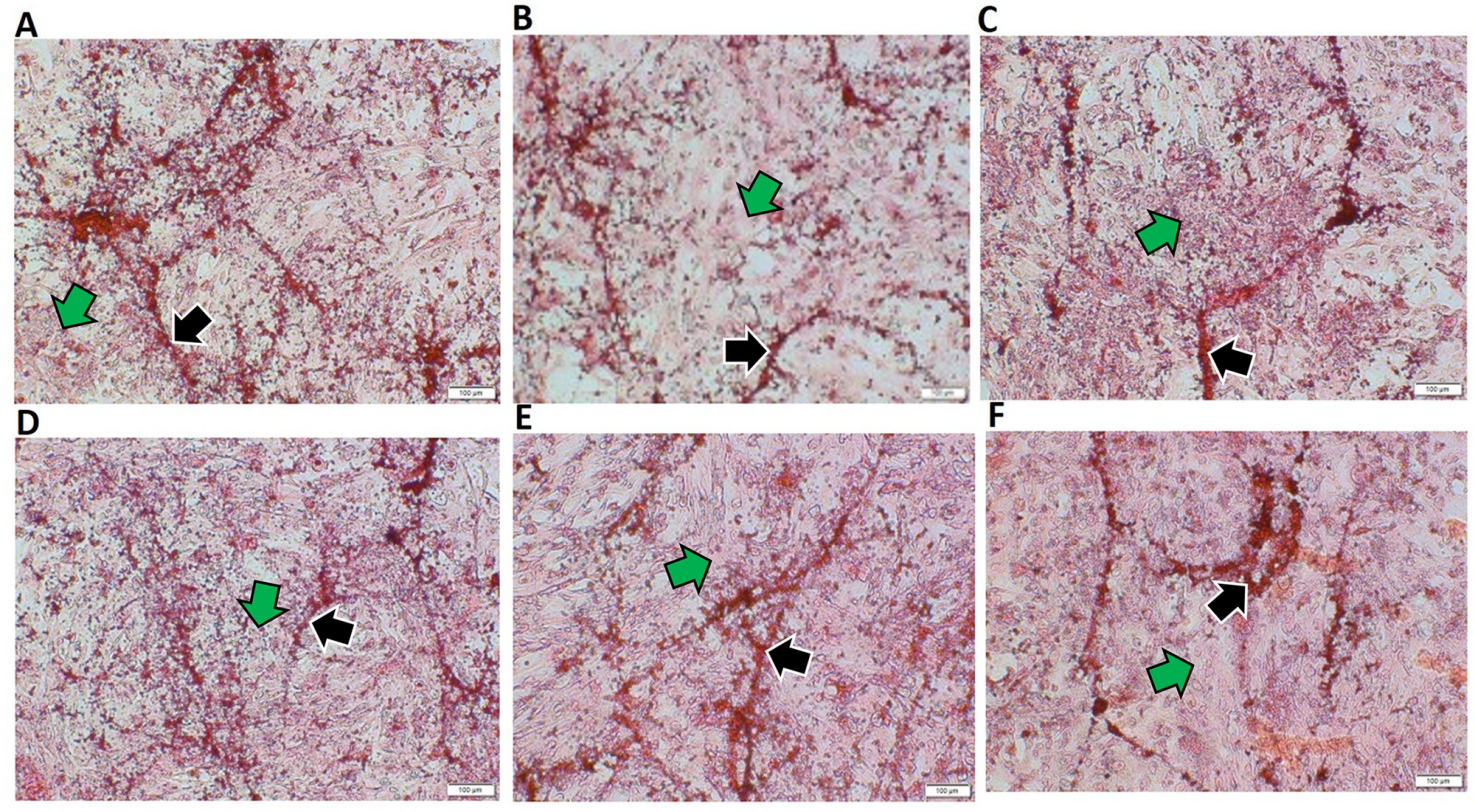
Representative Sirius red-stained light microscopy images of human osteoblasts treated with **(A)** Ct (DMSO vehicle treatment only), **(B)** BMP7 (Bone morphogenic protein 7), **(C)** EP1 (EP1 receptor antagonist group), **(D)** BMP7+EP1(the combination treatment of BMP7 with EP1 receptor antagonist), **(E)** EP2 (EP2 receptor agonist group), **(F)** BMP7+EP2 (the combination treatment of BMP7 with EP2 receptor agonist) after a duration of 21 days. Pink strands in the image marked by green arrows indicate less mature and newly formed collagen deposition and red strands marked black arrows denotes mature and well-organized collagen deposition. Scale bar 100 μm.

**Fig 6:**
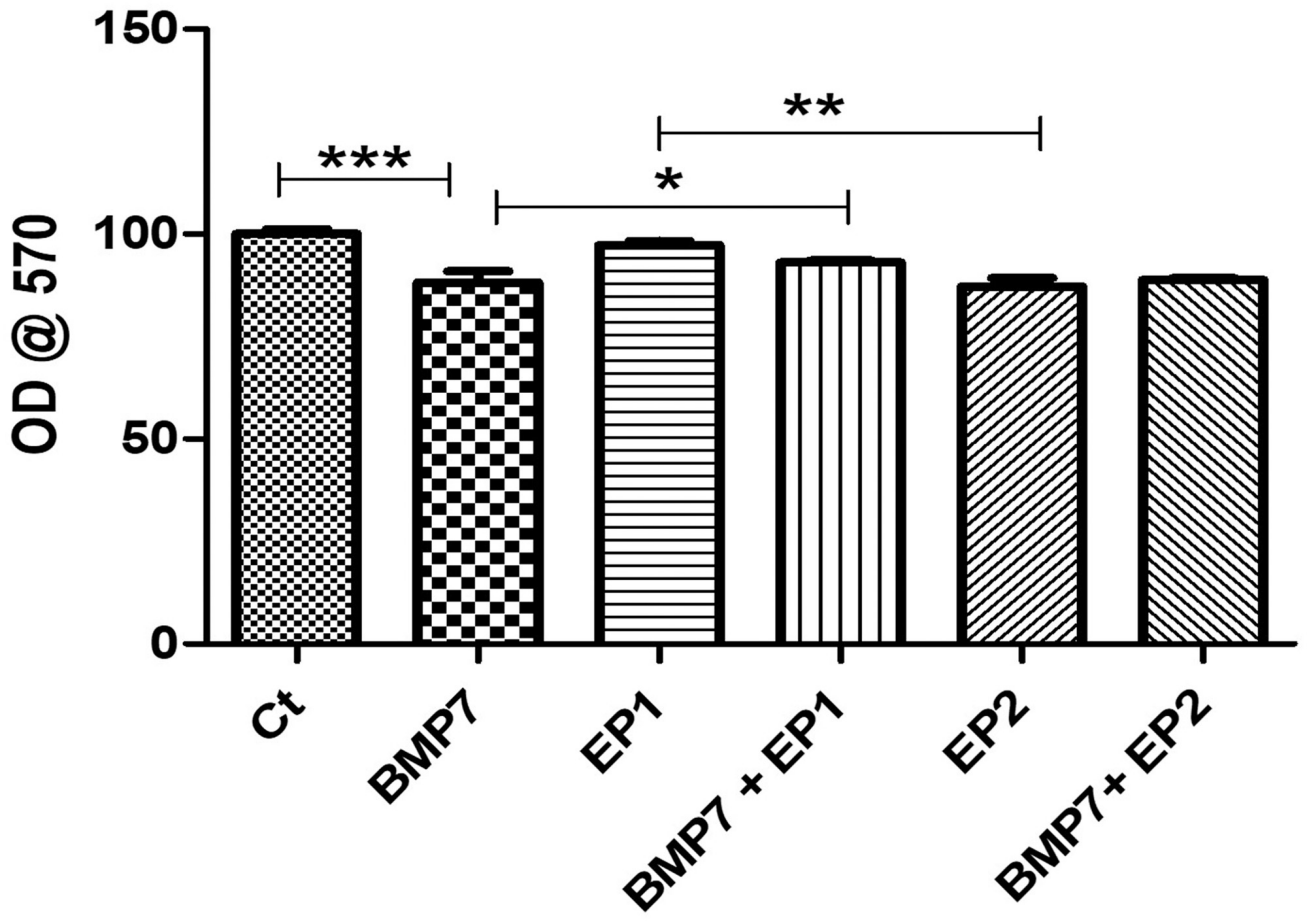
The graph represents the quantity of collagen synthesized by human osteoblasts across different treatment groups, including Ct (DMSO vehicle treatment only), BMP7 (Bone morphogenic protein 7), EP1 (EP1 receptor antagonist group), BMP7+EP1 (the combination treatment of BMP7 with EP1 receptor antagonist), EP2 (EP2 receptor agonist group), BMP7+EP2 (the combination treatment of BMP7 with EP2 receptor agonist) following a 21-days period. The findings are presented as mean ± standard deviation. Statistical analysis was done using ANOVA where *p<0.05, **p<0.01 and ***p<0.001 respectively.

Sirius red staining was used to examine the concentration and organization of collagen by the HOB cells in various treatment groups and was compared to the Ct. The greatest amount of collagen concentration as represented by both less mature collagen (green arrows) and mature collagen (black arrows) was observed in the Ct group and in the group receiving EP1 receptor antagonists, as shown in Figs 5 & 6. There was a statistically significant difference between these two groups and the other treatment groups (p<0.05). However, when the Ct group was compared to the EP2 receptor agonist group the difference was not statistically significant (p>0.05). Furthermore, the groups treated with BMP7 along with EP1 receptor antagonist exhibited a statistically significant increase in collagen concentration in comparison to the groups treated with BMP7 alone, EP2 receptor agonist alone, and the combination of BMP7 with EP2 receptor agonist (p<0.05).

Nevertheless, as stated previously the group treated with both BMP7 and EP1 receptor antagonist displayed significantly reduced collagen concentration (p<0.05) in comparison to the Ct and the group treated with EP1 receptor antagonist alone. Additionally, the group that received BMP7 treatment only showed a notably reduced collagen concentration level compared to the group that received the combination of BMP7 and EP1 receptor antagonist treatment (p<0.05).The EP2 receptor agonist alone group demonstrated the least collagen concentration, and this difference was statistically significant in comparison to the groups treated with EP1 receptor antagonist treatment alone, Ct and the combination of BMP7 with EP1 receptor antagonist (p<0.05).

### Alizarin red staining and calcium release

Alizarin red staining followed by calcium release absorbance measurement was done to access the level of calcium generated by human osteoblast cells under various treatments as mentioned in Table 1. Our experimental results (as shown in S1 & S2 Figs) revealed a statistically significant elevation in calcium concentration within the BMP7 treated groups as opposed to the Ct group, the EP1 receptor antagonist treated group, and the combination treatments involving BMP7 with either an EP1 receptor antagonist or an EP2 receptor agonist (p<0.05).

Remarkably, the only group exhibiting comparable results to the BMP7 alone treated group, was the group treated with EP2 receptor agonist alone demonstrating nearly identical results. However, the group treated with EP1 receptor antagonist alone displayed a significantly lower calcium concentration compared to both the BMP7 group, and the EP2 receptor agonist treatment group, nevertheless, EP1 receptor antagonist treated group exhibited a significantly augmented calcium concentration relative to the Ct group and the combination treatment group (p<0.05).

The Ct group exhibited a significantly lower calcium concentration compared to the groups treated with BMP7, EP1 receptor antagonist, and EP2 receptor agonist groups. However, it displayed a significantly higher calcium concentration compared to the combination treatment groups (p<0.05). The least calcium concentration was observed in the combination treatment groups, however when compared to each other, the difference in calcium concentration was not statistically significant (p>0.05).

### Fourier Transform Infrared Spectroscopy (FT-IR)

**Fig 7:**
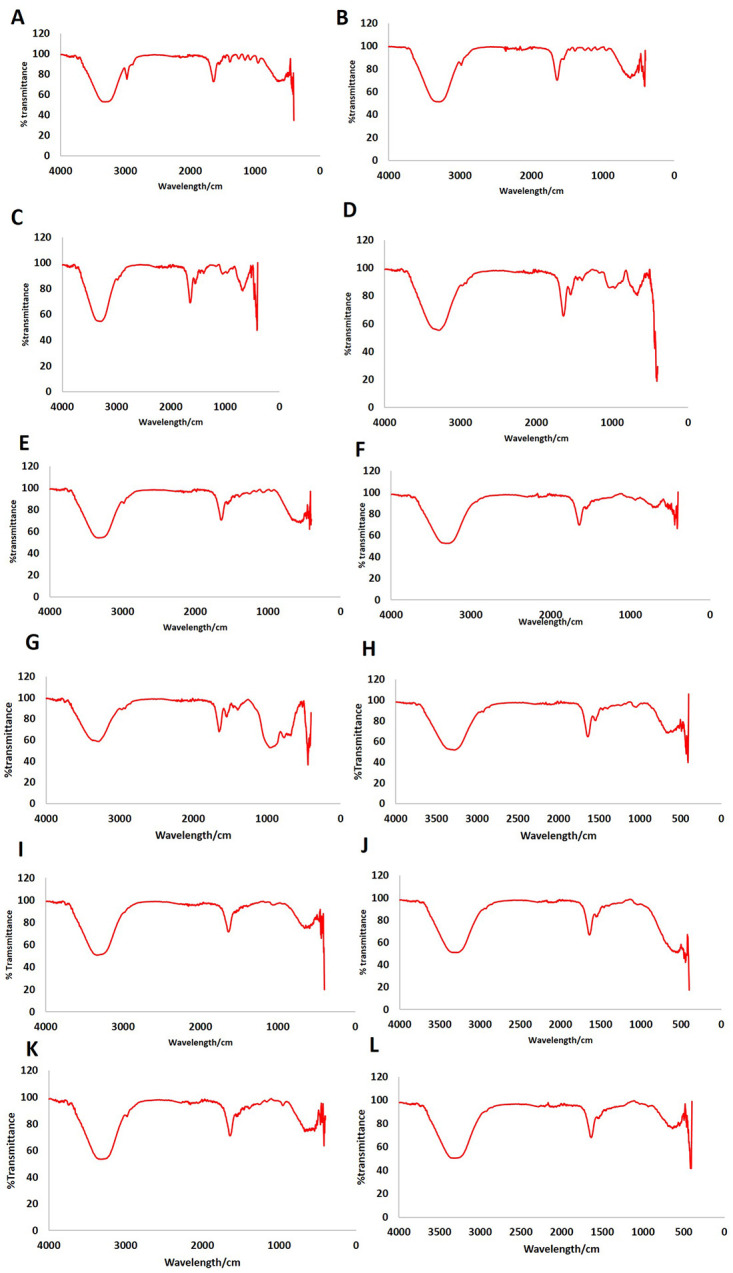
Representative images of the Fourier Transform Infrared Spectroscopy (FT-IR) graphs utilized for the examination of mineral concentration and collagen parameters in each sample. **(A)** Ct: OS-; **(B)** Ct: OS+; **(C)** BMP7: OS-; **(D)** BMP7: OS+; **(E)** EP1: OS-; **(F)** EP1: OS+; **(G)** BMP7+EP1: OS-; **(H)** BMP7+EP1: OS+; **(I)** EP2: OS-;**(J)** EP2: OS+; **(K)** BMP7+EP2: OS-; **(L)** BMP7+EP2: OS+ respectively.

**Fig 8:**
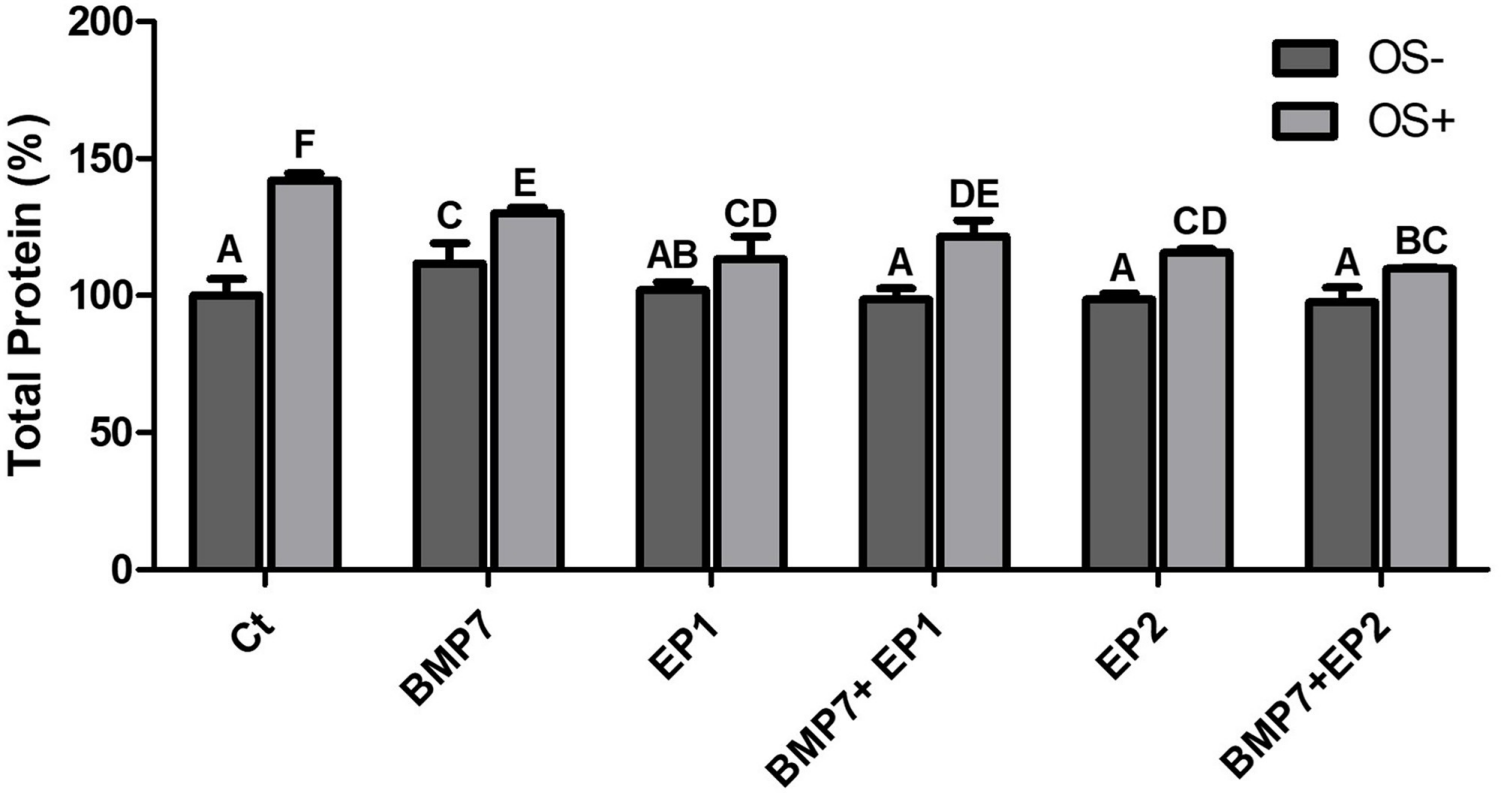
Bar graph demonstrating the total protein % produced by the human osteoblasts among the different treatment groups as shown in Table 1 without osteogenic media induction (OS-) and with osteogenic media induction (OS+) after the period of 21 days. The findings are presented as mean ± standard deviation and statistical analysis was conducted through ANOVA. Groups sharing identical alphabets above each bar indicate no significant statistical differences.

**Fig 9:**
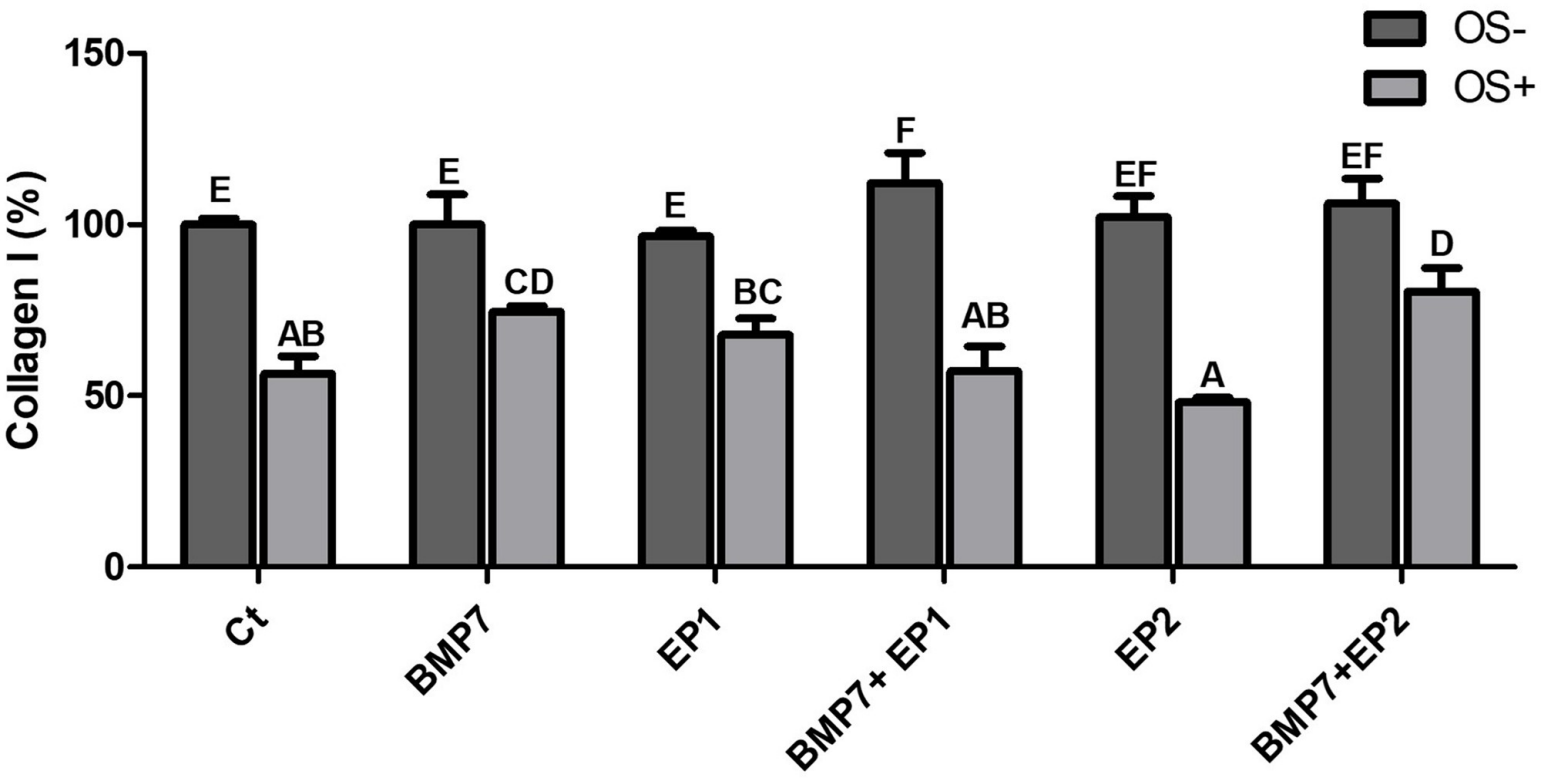
Bar graph demonstrating the total collagen I concentration % produced by the human osteoblasts across diverse treatment groups as shown in Table 1 without osteogenic media induction (OS-) and with osteogenic media induction (OS+) after the period of 21 days. The findings are presented as mean ± standard deviation and statistical analysis was conducted through ANOVA. Groups sharing identical alphabets above the bar indicate no significant statistical differences.

**Fig 10:**
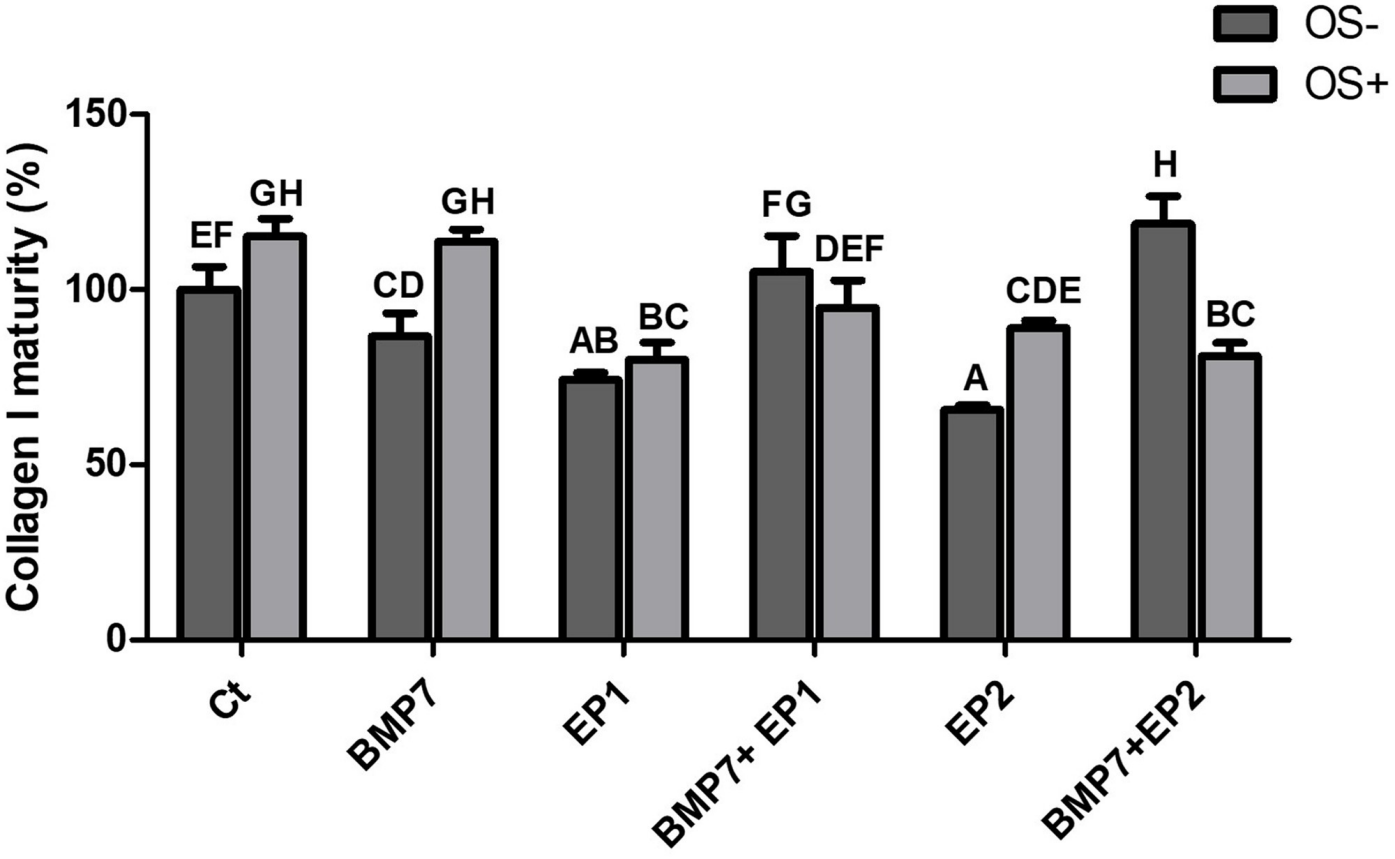
Bar graph illustrating the mature type I collagen concentration % produced by the human osteoblasts across various treatment groups as shown in Table 1 without osteogenic media induction (OS-) and with osteogenic media induction (OS+) after the period of 21 days. The findings are presented as mean ± standard deviation and statistical analysis was conducted through ANOVA. Groups sharing identical alphabets above the bar indicate no significant statistical differences.

**Fig 11:**
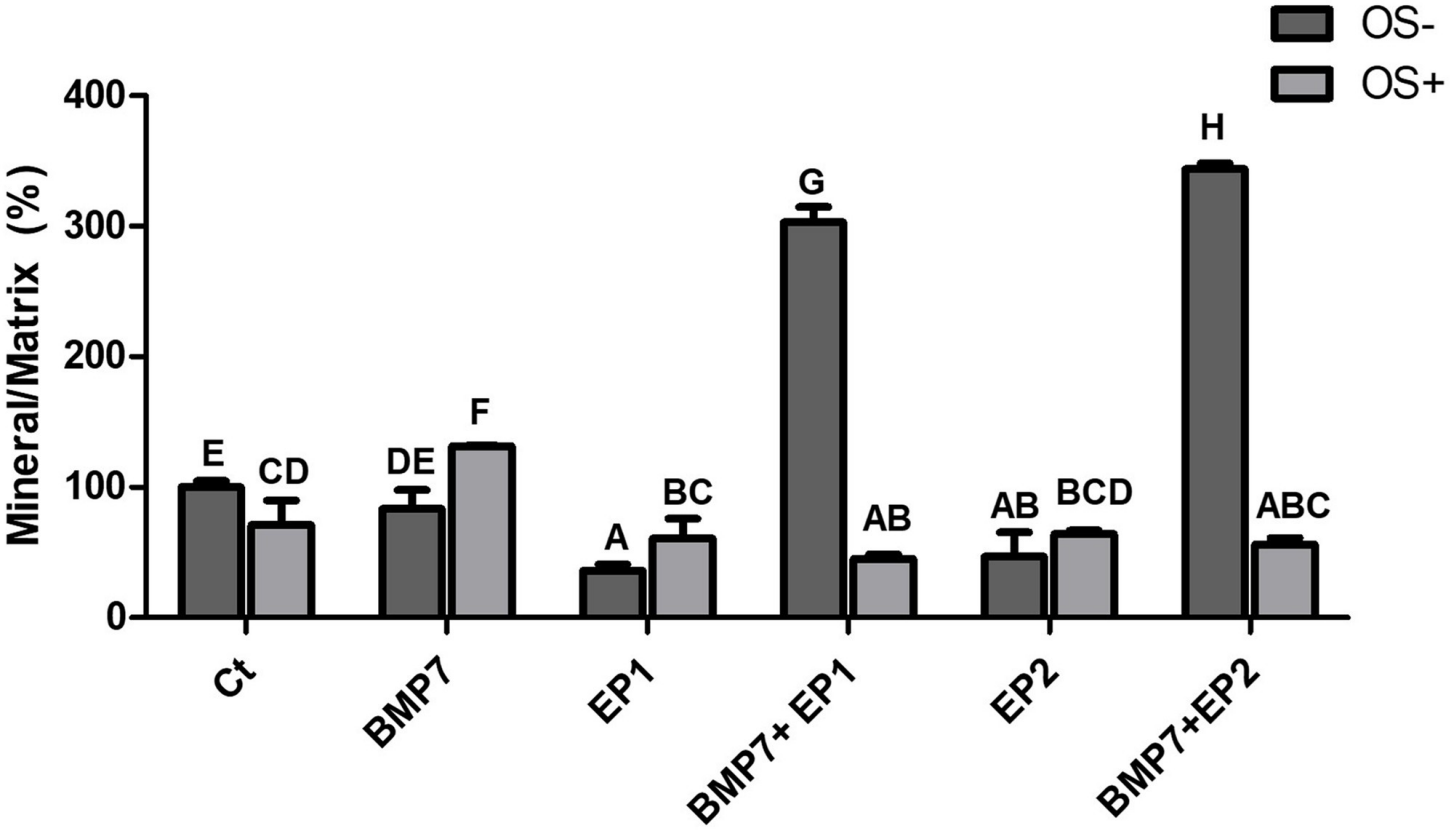
Bar graph demonstrating the mineral/ matrix deposition % by the human osteoblasts under different treatment groups as shown in Table 1 without osteogenic media induction (OS-) and with osteogenic media induction (OS+) after the period of 21 days. The findings are presented as mean ± standard deviation and statistical analysis was conducted through ANOVA. Groups sharing identical alphabets above the bar indicate no significant statistical differences.

In this experiment the FT-IR machine was employed to acquire data (as shown in Fig 7) on various parameters among the different treatment groups as mentioned above. The data obtained from this study (as shown in Figs 8–11) were used to determine the total protein concentration, the total concentration of collagen synthesized by different treatment groups, the concentration of mature collagen, and the mineral content produced by the different treatment groups. The FT-IR analysis facilitated and provided insights into the distinctive molecular compositions and characteristics within each experimental condition.

### Total protein concentration (%)

After gathering the FT-IR data the results were compared among the different groups with particular attention given to know the potential differential impact on mature versus immature osteoblasts. Initially, the average values of total protein concentration were accessed prior to categorizing the groups based on the treatment with osteogenic media (OS+) and a group that was not induced with osteogenic media (OS-). A significantly higher average protein concentration was seen in the Ct group, and the group treated with BMP7 alone, when compared to other groups (p<0.05), however the difference between those two groups was not significant (p>0.05). The group treated with the combination of BMP7 with EP1 receptor antagonist as stated earlier has shown significantly less protein concentration than the Ct group, and the group treated by BMP7 alone, however this combination of BMP7 with EP1 receptor antagonist has shown a significantly higher protein concentration when compared to the other combination treatment group of BMP7 with EP2 receptor agonist (p<0.05). The least total protein concentration was seen in the groups treated with EP1 receptor antagonist alone, EP2 receptor agonist alone, and the combination treatment of BMP7 with EP2 receptor agonist, and the difference between those groups was not statistically significant (p>0.05).

Next the treatment groups were divided into groups treated with osteogenic medium, and groups that were not. All the treatment groups that were treated with the osteogenic media exhibited significantly higher total protein concentration compared to their counterparts that didn’t receive the osteogenic medium treatment (p<0.05). Among all the groups, the Ct group that was treated with the osteogenic medium has shown the highest protein concentration compared to all other groups (p<0.05). The second highest total protein concentration was observed in the group treated with BMP7 alone in the osteogenic medium, in which the total protein concentration was significantly higher than all other groups (p<0.05), except for the Ct group with the osteogenic medium. The groups treated with EP1 receptor antagonist alone, EP2 receptor agonist, and the combination treatments all have exhibited similar results when compared to each other, when all the treatments were grown in an osteogenic media (p>0.05), and all of those groups have showed a significantly less total protein concentration when in contrast compared to the groups treated with Ct, and BMP7 treatment alone (p<0.05) when cultured in the same osteogenic media.

When comparing the treatment groups without induction with osteogenic media it was seen that the group treated with BMP7 alone has shown a significant increase in the total protein concentration compared to the other groups that were also grown in a non-osteogenic medium (p<0.05); this group has showed almost similar results to the groups treated with EP1 antagonist, EP2 agonist, and the combination of BMP7 with EP2 agonist with osteogenic medium. Aside from the group treated with BMP7 alone, all other groups exhibited a nonsignificant difference between each other when they were not grown in an osteogenic medium (as shown in Fig 8).

### Total collagen I concentration (%)

The total collagen I concentration % was calculated using FT-IR analysis results. The average collagen I concentration % obtained from the HOB cells grown in an osteogenic media (OS+) with various treatment groups as indicated in Table 1 were compared to HOB cells grown in the same treatment groups but without the osteogenic media (OS-). (Fig 9) shows that groups treated with the combination of BMP7 with EP2 agonist exhibited significantly higher total collagen I concentration % in comparison to the other groups (p<0.05) except for the BMP7 alone treatment group, where the difference was not statistically significant (p>0.05). The group treated with BMP7 alone showed significantly higher total collagen I concentration when compared to the groups treated with EP2 agonist alone, and the Ct group (p<0.05) but the difference was not significant between this group and the group treated with EP1 antagonist, and the combination treatments of BMP7 with EP1 antagonist, and BMP7 with EP2 agonist (p>0.05). The least total collagen I concentration % was noticed in the group treated with EP2 agonist alone, but the difference was significant only when compared to either BMP7 treatment alone or the combination treatment of BMP7 with EP1 antagonist, and BMP7 with EP2 agonist (p<0.05).

Next, when the total collagen I concentration % was compared among the groups that were grown in an osteogenic medium and the groups that were not induced with osteogenic media the groups that were not grown in an osteogenic medium exhibited a significant increase in the total collagen I concentration % compared to their counterparts that were grown in an osteogenic medium (p<0.05). The highest collagen I concentration was seen in the combination treatment of BMP7 with EP1 antagonist that was not grown in an osteogenic medium, but the difference was not significant when compared to the groups that were treated with EP2 agonist alone, and the combination of BMP7 with EP2 agonist that were grown in the same medium. All other groups exhibited similar results to each other, and the differences between them was not statistically significant.

As stated earlier, the groups that were grown in an osteogenic medium exhibited significantly less total collagen I concentration compared to their counterparts that were not. When comparing the collagen concentration between the groups that were grown in an osteogenic medium it was shown that the combination of BMP7 with EP2 agonist has shown a significantly higher collagen I concentration when compared to the other groups that were treated in the osteogenic medium (p<0.05), except for the group that was treated with BMP7 alone (p>0.05). For the group that was treated with BMP7 alone, it was shown that the total collagen I concentration was significantly higher than the Ct group, the EP2 agonist group, and the combination of BMP7 with EP1 antagonist group (p<0.05). However, for the group that was treated with EP1 antagonist alone, it was shown that it exhibited a significantly higher collagen I concentration when compared to the group treated with EP2 agonist alone (p<0.05). The least collagen I concentration was seen in the group treated with EP2 agonist alone, and the difference was significant when compared to the groups treated with BMP7 alone, the EP1 antagonist group, and the combination of BMP7 with EP2 agonist (p<0.05).

### Mature collagen I concentration (%)

The next stage was to measure the concentration of mature collagen I amongst the test groups. When examining the average concentration between the groups, it was seen that the highest mature collagen I concentration was seen in the group treated with Ct in which all other groups had a significantly lower concentrations when compared to this group (p<0.05) (as shown in Fig 10). The group that was treated with BMP7 alone has exhibited the second highest mature collagen I concentration aside from the group treated with Ct. The BMP7 group had a significantly higher collagen I concentration compared to the groups treated with EP1 antagonist, and EP2 agonist (p<0.05), however, the discrepancy did not reach statistical significance when compared to the combination treatment groups (p>0.05). The combination treatments have exhibited similar results in which they were significantly higher than the EP1 antagonist group, and the EP2 agonist group, and significantly lower than Ct group (p<0.05) the lowest concentration of mature collagen was seen in the groups treated with EP1 antagonist, and EP2 agonist, in which the mature collagen I concentration showed significantly lesser than the other group (p<0.05).

Next mature collagen I concentration was compared between cells that were induced with osteogenic media (OS+) and the cells that were not induced with osteogenic media (OS-).The data showed that the Ct treatment group, the BMP7 group, and the EP2 agonist groups exhibited a significant increase in mature collagen I concentration in the cells that were treated in the osteogenic medium (OS+) compared to their counterparts that were not treated in the osteogenic medium (OS-) (p<0.05). The cells that were treated with the combination of BMP7 with EP2 agonist exhibited a significantly lower mature collagen I concentration in the group that was treated in the osteogenic medium compared to their counterpart in the group that was not treated in the osteogenic medium (p<0.05). The groups treated with the combination of BMP7 with EP1 antagonist, and EP1 antagonist treatment alone exhibited no significant difference in mature collagen I concentration irrespective of the cells being treated in an osteogenic environment or not (p>0.05).

The highest concentration of mature collagen I was seen in the combination treatment of BMP7 with EP2 agonist group that was not treated in the osteogenic medium, and the difference was significant in all groups (p<0.05), except when compared to the Ct group, and the BMP7 group that were treated in the osteogenic medium. The lowest mature collagen I concentration was seen in the group that was treated with EP2 agonist alone without the osteogenic medium, and the difference was statistically significant when compared to all the groups (p<0.05) except when compared to the group that was treated with EP1 antagonist alone excluding the osteogenic medium (p>0.05).

### Mineral by matrix ratio %

The ultimate focus of our investigation centered on mineral concentrations within the diverse treatment groups. When the average of all the groups were gathered and normalized with the Ct group it was evident that the combination treatment groups exhibited the highest minerals concentration among all the other treatment groups (as shown in Fig 11). The group that was treated with the combination of BMP7 with EP2 agonist has shown a significant increase in the mineral content when compared to all other groups (p<0.05). Similarly, the combination of BMP7 with EP1 antagonist has also shown a significant increase in the mineral content when compared to the other groups (p<0.05), except when compared to the BMP7 with EP2 agonist which showed significantly lower mineral concentration than the latter group (p<0.05). In contrast, the group treated with BMP7 alone exhibited significantly less mineral concentration when compared to the combination treatment groups (p<0.05), however, when compared to the Ct group, the EP1 antagonist, and EP2 agonist groups, the BMP7 treatment alone exhibited significantly higher mineral concentrations (p<0.05). The least mineral concentrations were noticed in the groups treated with EP1 antagonist alone, and EP2 agonist alone (p<0.05).

When assessing the influence of the osteogenic medium on the mineral content of the treatment groups, it was evident that the Ct group, and the combination treatment groups when treated without osteogenic medium (OS-) exhibited a significantly higher mineral concentration comparted to their counterparts that were treated with osteogenic medium (OS+) (p<0.05). In the groups treated with BMP7 alone, and EP1 antagonist alone, it was noted upon treating the cells with the osteogenic medium, there was a significant increase in the mineral concentration compared to their counterparts not treated in the osteogenic medium (p<0.05). However, for the group treated with EP2 agonist alone, no difference in the mineral concentration was noted whether the cells were treated with osteogenic media or not (p>0.05).

## Discussions

This study was conducted with the focus to assess the effect of the combination treatments of Prostaglandin EP2 receptor agonist or an EP1 receptor antagonist alone with a low dose BMP7 mimicking in vivo bone healing environment using human osteoblast cell line in vitro. In this study 6 treatment groups were studied as shown in Table 1. The first group was the control group (Ct) in which the cells were treated by DMSO in all the experiments. The second group was the group treated with BMP7 alone. The third and fourth groups were the groups treated with EP1 receptor antagonist alone, and the combination treatment of BMP7 with EP1 receptor antagonist respectively. The final two groups were the cells treated with EP2 receptor agonist, and cells under the treatment EP2 agonist coupled with BMP7. Our hypothesis was that utilizing the combinations of two anabolic drugs BMP7 with either Prostaglandin EP2 receptor agonist or Prostaglandin EP1 receptor antagonist simultaneously would lead to an increase in cellular migration and thus reduction of gap in the scratch wound assay, increase collagen concentration, and elevated mineral levels within the matrix compared to the single drug treatment alone. The overall aim was to explore whether the synergistic effects of the combination treatments could enable a reduction in the dosage of the respective drugs, hence reducing the negative systemic consequences when used in tandem.

The use of EP1 antagonist in this study was due to the evidence provided by Feigenson et al. in 2020 [32], in which they conducted an experiment on rats and the findings indicated that early-stage administration of an EP1 receptor antagonist to wild-type mice led to increased osteoblast differentiation and enhanced fracture callus formation. The decision regarding the choice of using EP2 receptor agonist was influenced by the research conducted by Paralkar et.al. in 2003 [33], in their study they injected EP2 receptor agonist into a fracture area on rats and concluded that EP2 receptor agonists stimulates new bone formation on trabecular, endocortical, and periosteal surfaces and enhances fracture healing. Finally, in a review done by Cecchi et al. in 2015 [34], the signaling pathway of BMP7 was discussed in detail, along with all its benefits on bone healing. They also included clinical trials used to compare between the use of autogenous bone grafting, and the use of collagen infused with BMP7 to treat nonunion, and they concluded that autogenous bone grafting exhibited similar results in healing when compared to collagen infused with BMP7.

The wound healing assay results showed that the Ct group followed by BMP7 treatment alone demonstrated the highest percentage of wound closure after the scratch was made in the HOB cell monolayer than the combination treatment of BMP7 with prostaglandin EP1 receptor antagonist or BMP7 with prostaglandin EP2 receptor agonist compare to the single drug treatment. The observation that BMP7 treatment alone demonstrated increased wound closure than the combination treatment is consistent with the previous research that BMP7 stimulates cell migration and extracellular matrix deposition during wound repair process through various signaling pathways including the SMAD and MAPK pathways, which is crucial in regulating key cellular response involved in wound healing [34, 35]. The stimulation of these pathways via BMP7 likely contribute for its efficiency in inducing wound closure in HOB cell. This tells us that each treatment on its own is very effective and when combining the BMP7 with the other two treatments, there was a non-complementary effect of the treatment on the osteoblastic cells. The reduced efficacy of combination treatments containing the prostaglandin EP1 receptor antagonist or EP2 receptor agonist raises questions about the interaction between BMP7 signaling and prostaglandin pathways during wound healing. Prostaglandins are lipid mediators generated from arachidonic acid metabolism that have various effects on inflammation, vascular permeability, and tissue repair. While the exact mechanisms underlying BMP7 prostaglandin signaling interactions are unknown, it is possible that crosstalk occurs at multiple levels, including receptor activation, intracellular signaling cascades, and gene expression regulation. One possible explanation for the reduced wound healing response reported with combination therapies is the regulation of inflammatory responses. Prostaglandins are reported to cause pro-inflammatory effects (5,11) which might minimize the regenerative actions of BMP7. Furthermore, the balance of pro-inflammatory and anti-inflammatory signals is critical for coordinating various stages of wound healing and disrupting this balance may hamper tissue healing processes. Thus, manipulating the BMP7 and prostaglandin pathways simultaneously may imbalance the healing process resulting in inferior wound closure. While prostaglandin EP1 receptor antagonist and EP2 receptor agonist are likely to modulate different downstream signaling pathways their combined effects on BMP7 mediated wound healing is complex and may involve elaborate regulatory mechanisms. Thus, further research into the molecular mechanisms behind these interactions is needed for the development of more effective wound healing treatments. Nevertheless, as bone regeneration is a multicellular phenomenon there might be other factors which might also influence human osteoblast cellular migration.

To check the collagen production by osteoblasts, the samples were stained with Sirius Red stain. In this experiment, it was observed that the highest collagen production was observed in the cells treated with Ct, and EP1 antagonist alone. This result is consistent with a previous study done by Zhang et al. in 2011 [36] in which they examined mice that lack EP1 receptors, and it was observed that these mice exhibit a higher collagen concentration than their wild type counterparts.

Another interesting finding that our results highlights is the interplay between BMP7 and prostaglandin E2 signaling pathways in collagen production, a critical element for bone growth and repair. Our findings shows that the combination of BMP7 with an EP1 antagonist results in significantly higher collagen levels than the different other groups treated with BMP7 alone, EP2 agonist alone, and the combination of BMP7 with EP2 agonist. Our results are consistent with the previous findings demonstrating the critical role of BMP7 in bone formation [34]. However, the synergistic effects observed between BMP7 and EP1 receptor antagonist is a novel new insight in bone regeneration process. Inhibition of the EP1 receptor increases the osteogenic effects of BMP7 most likely via counteracting EP1 mediated inhibition of osteoblast differentiation and bone formation. This finding highlights the complex cross talk between BMP7 and PGE2 signaling pathways. Our findings imply that inhibiting the EP1 receptor enhances the osteogenic effects of BMP7, resulting in increased collagen synthesis. This synergistic effect could be related to the complex interplay between the BMP and PGE2 signaling pathways. EP1 receptor activation has been associated to inhibiting osteoblast development and bone formation [36]. By blocking the EP1 receptor the bone formation pathway might have been mitigated thereby increasing the osteogenic activity of BMP7. In contrast, the lack of significant increase in collagen formation observed in groups treated with BMP7 alone or in combination with the EP2 agonist highlights the intricacy of PGE2 signaling in bone metabolism. While EP2 receptor activation has been linked to anabolic effects on bone [37, 38], our data indicate that its stimulation in combination with BMP7 may not produce synergistic benefits as those observed with EP1 antagonist. Further investigation into the underlying mechanisms of BMP7 and EP1 antagonist combination is necessary. Understanding the downstream signaling pathways impacted by this combination may provide valuable insights into wound repair mechanisms.

To estimate the amount of calcium released from the osteoblasts, the human osteoblast cells underwent staining using the alizarin red and the calcium released was measured at absorbance 405 (as shown in S1 & S2 Figs). The result from this experiment showed the largest calcium deposition in the groups treated with BMP7 alone and EP2 agonist alone. This result was supported by another study done by Li et al. 2003 [38] in which it was reported that activation of EP2 receptors in osteoblasts can enhance the expression of genes and proteins involved in calcium transport and bone matrix mineralization. For the group that was treated with EP1 antagonist, it was observed that this group exhibited significantly less production of calcium when compared to the groups treated with BMP7 alone, and EP2 agonist alone. However, there was also a significantly higher calcium deposition compared to the groups treated with Ct, and the combination treatments. This indicates that each treatment independently stimulates calcium production effectively. Nevertheless, as observed in the previous experiments the combination treatments showed the lowest calcium concentrations among all groups, suggesting antagonistic effects on calcium deposition between them.

The final step of this study involved the FI-TR analysis to access various parameters such as the total protein concentration, the total concentration of collagen produced, the concentration of mature collagen, and the mineral to matrix content. Groups treated with Ct and BMP7 alone exhibited the highest total protein content. Notably, in our study the EP1 receptor antagonist appeared to downregulate the osteoblast’s ability to produce proteins, while EP2 receptor agonist negatively impacted protein production which is consistent and coincides with the study by Alander and Raisz et al. 2006 [39]. Interestingly, in our study we found that although BMP7 treatment alone could stimulate osteoblast to produce more proteins combining BMP7 with EP1 receptor antagonist or EP2 receptor agonist reduced the ability of the osteoblasts to produce more proteins.

For the collagen type I production, the highest concentration was seen in the combination treatment of BMP7 with EP2 receptor agonist. In this experiment the data indicated that the combination of BMP7 with EP2 receptor agonist seemed to complement each other and did not antagonize each other’s function. However, the effect of EP2 receptor agonist alone on the cells seemed to downregulate the cellular potential to produce collagen type I. The result that we observed in our study is contrary to a study by Li et al. 2003 [38] in which it was observed that EP2 receptor agonists can stimulate the production of collagen type I in osteoblasts indicating a complex interplay between the treatment pathways.

In the final experiment we aimed to investigate the extent of mineral deposition per matrix. Interestingly the results obtained from this study showed that the highest mineral deposition per matrix were among the combination treatment groups specifically when BMP7 was combined with either EP1 receptor antagonist or EP2 receptor agonist the resulting matrix appeared to be more mature and harder compared to other treatment groups. This finding highlights a possible synergistic effect between BMP7 and EP receptors on matrix maturation. This could be due to the EP1 antagonist and EP2 agonist, as the alizarin red staining experiment revealed that they stimulate calcium release by osteoblasts. When EP1 antagonists or EP2 agonists were employed alone, they had the lowest mineral concentration per matrix. The group treated with BMP7 alone appeared to contain more minerals per matrix concentration than the Ct group. This is consistent with the findings of Cecchi et al. in 2015 [34], who concluded that BMP7 enhances osteoblast matrix deposit while also increasing mineralization of the set matrix. Taken together the findings suggests that combining BMP7 with EP receptors could be a promising approach for enhancing bone matrix maturation and mineralization thereby providing new insights into bone regeneration strategies.

Nonetheless, further future studies are required to know the effects of this combination therapy across different cell types and in vivo settings. Additionally, the results that are presented in this study may not precisely reflect the behavior of EP1 receptor antagonist and EP2 receptor agonist in vivo. Despite these limitations, this is the first study demonstrating individual drug’s efficacy in accelerating human osteoblast cell regeneration and comparing it to the combination treatment of BMP7 with EP1 receptor antagonist or EP2 receptor agonist. However, the co-administration of these drugs reduced the human osteoblast healing, maturation and concentration of the collagen compared to the BMP7 treatment alone and did not produce the anticipated complementary effect that was initially hypothesized.

## Conclusion

In summary our in vitro study using human osteoblast cell line suggests that the combination of Prostaglandin EP2 receptor agonist and an EP1 receptor antagonist, when combined with BMP7 significantly impede human osteoblast healing, the maturation and concentration of collagen compared to the BMP7 treatment alone.

## Supporting information

S1 Fig Illustrative microscopic images showing human osteoblast cells subjected to various treatments for 21 day period under osteogenic condition, followed by alizarin red staining manifesting as a concentrated orange color marked with yellow arrow: **(A)**: Ct (DMSO vehicle treatment only), **(B)**: BMP7 (Bone morphogenic protein 7 treatment), **(C)**: EP1 (EP1 receptor antagonist group), **(D)**: BMP7+EP1 (the combination treatment of BMP7 with EP1 receptor antagonist), **(E)**: EP2 (EP2 receptor agonist group) and **(F)**: BMP7+EP2 (the combination treatment of BMP7 with EP2 receptor agonist) respectively. Scale bar 50 μm.
(TIF)

S2 Fig Bar graph illustrating the calcium release absorbance levels by human osteoblasts after treatment with Ct (DMSO vehicle treatment only), BMP7 (Bone morphogenic protein 7), EP1 (EP1 receptor antagonist group), EP2 (EP2 receptor agonist group), BMP7+EP1 (the combination treatment of BMP7 with EP1 receptor antagonist) and BMP7+EP2 (the combination treatment of BMP7 with EP2 receptor agonist) respectively for 21 days period under osteogenic condition. The findings are presented as mean ± standard deviation. Statistical analysis was done using ANOVA where *p<0.05 and ***p<0.001 respectively.
(TIF)

## Abbreviations

BMP7: Bone Morphogenetic Protein 7
Ct: Control
DMEM: Dulbecco’s Modified Eagle Media
DMSO: Dimethyl Sulfoxide
EP1: Prostaglandin EP1 receptor
EP2: Prostaglandin EP2 receptor
FT-IR: Fourier-Transform Infrared Spectroscopy
h: hours
HOB: fetal Human osteoblast/ human osteoblast
ng/ml: Nanogram per milli liter
OS-: DMEM media without osteogenic induction
OS+: DMEM media with osteogenic induction
PFA: Paraformaldehyde
PGE2: prostaglandin E2
