# Supplementary figures and images for: Impact of Bone Morphogenetic Protein 7 and Prostaglandin receptors on osteoblast healing and organization of collagen
Source: PLoS One. 2024 May 16;19(5):e0303202. doi: 10.1371/journal.pone.0303202 (PMC11098345; doi:10.1371/journal.pone.0303202)

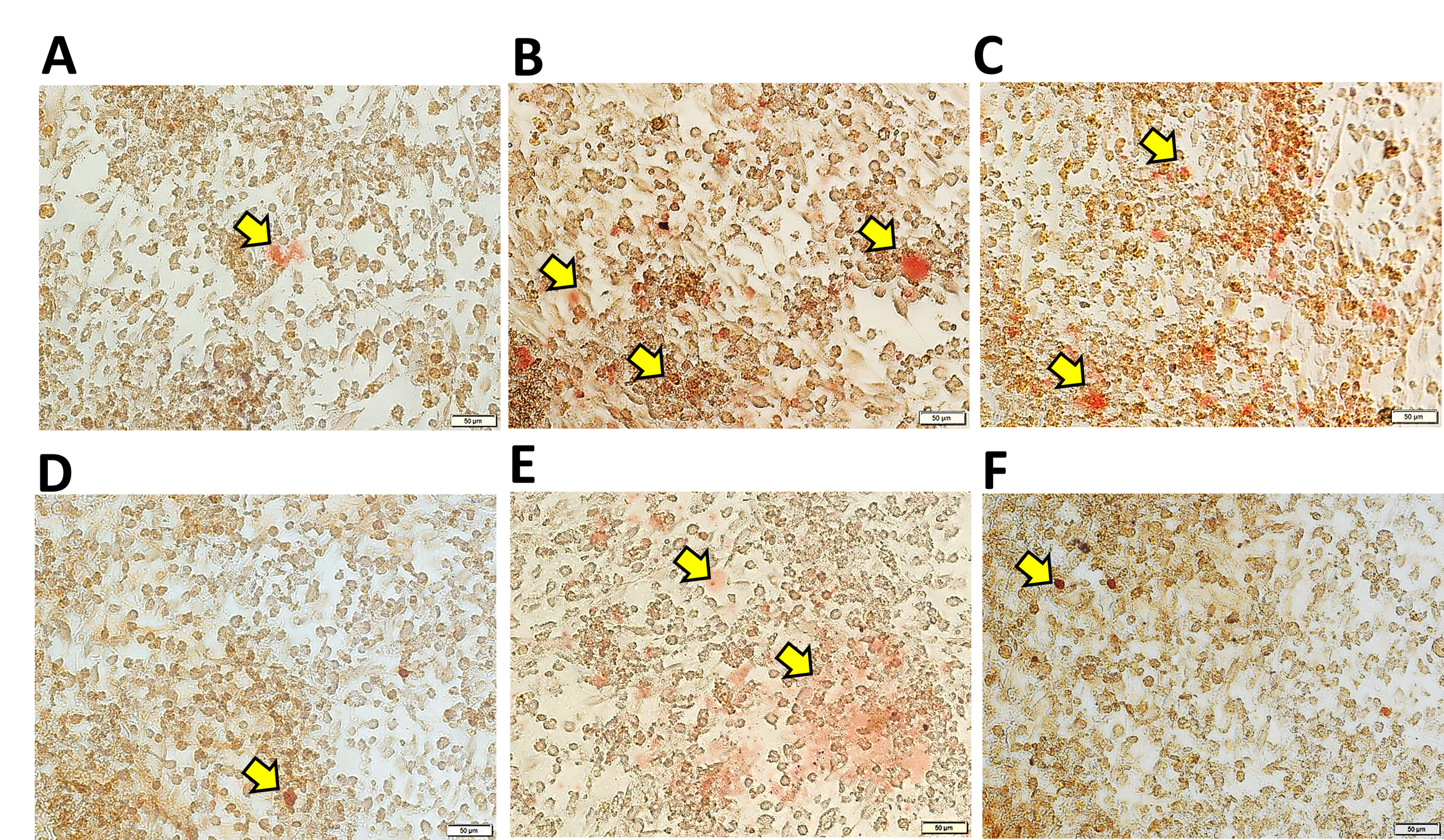

Supplement: S1 Fig — Illustrative microscopic images showing human osteoblast cells subjected to various treatments for 21 day period under osteogenic condition, followed by alizarin red staining manifesting as a concentrated orange color marked with yellow arrow: (A): Ct (DMSO vehicle treatment only), (B): BMP7 (Bone morphogenic protein 7 treatment), (C): EP1 (EP1 receptor antagonist group), (D): BMP7+EP1 (the combination treatment of BMP7 with EP1 receptor antagonist), (E): EP2 (EP2 receptor agonist group) and (F): BMP7+EP2 (the combination treatment of BMP7 with EP2 receptor agonist) respectively. Scale bar 50 μm. (TIF) [file pone.0303202.s001.tif]

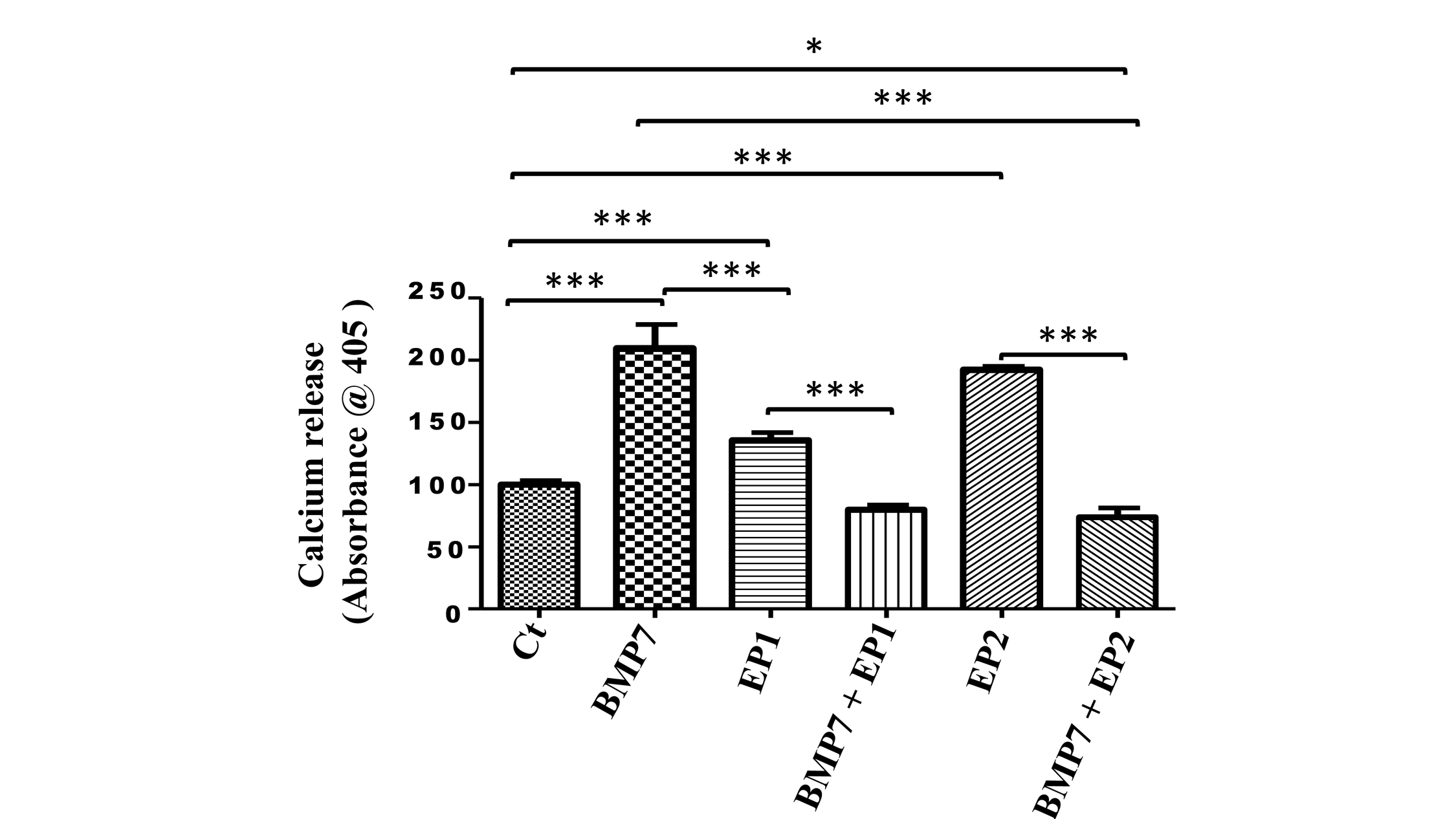

Supplement: S2 Fig — The findings are presented as mean ± standard deviation. Statistical analysis was done using ANOVA where *p<0.05 and ***p<0.001 respectively. (TIF) [file pone.0303202.s002.tif]
